# Identification and Validation of a Novel Multiomics Signature for Prognosis and Immunotherapy Response of Endometrial Carcinoma

**DOI:** 10.1155/2022/8998493

**Published:** 2022-10-15

**Authors:** Zhicheng Wu, Qiu Wang, Xiuqing Liu, Jiantong Zheng, Ling Ji, Xiaofeng Li

**Affiliations:** ^1^Department of Laboratory Medicine, Peking University Shenzhen Hospital, Shenzhen, China; ^2^Department of Laboratory Medicine, Shenzhen Second People's Hospital, Shenzhen, China; ^3^Shenzhen Dymind Biotechnology Company Limited, Shenzhen, China

## Abstract

**Purpose:**

Cancer development and immune escape involve DNA methylation, copy number variation, and other molecular events. However, there are remarkably few studies integrating multiomics genetic profiles into endometrial cancer (EC). This study aimed to develop a multiomics signature for the prognosis and immunotherapy response of endometrial carcinoma.

**Methods:**

The gene expression, somatic mutation, copy number alteration, and DNA methylation data of EC were analyzed from the UCSC Xena database. Then, a multiomics signature was constructed by a machine learning model, with the ROC curve comparing its prognostic power with traditional clinical features. Two computational strategies were utilized to estimate the signature's performance in predicting immunotherapy response in EC. Further validation focused on the most frequently mutant molecule, ARID1A, in the signature. The association of ARID1A with survival, MSI (Microsatellite-instability), immune checkpoints, TIL (tumor-infiltrating lymphocyte), and downstream immune pathways was explored.

**Results:**

The signature consisted of 22 multiomics molecules, showing excellent prognostic performance in predicting the overall survival of patients with EC (AUC = 0.788). After stratifying patients into a high and low-risk group according to the signature's median value, low-risk patients displayed a greater possibility of respond to immunotherapy. Further validation on ARID1A suggested it could induce immune checkpoints upregulation, promote interferon response pathway, and interact with Treg (regulatory T cell) to facilitate immune activation in EC.

**Conclusion:**

A novel multiomics prognostic signature of EC was identified and validated in this study, which could guide clinical management of EC and benefit personalized immunotherapy.

## 1. Introduction

As the most prevalent gynecologic malignancy, endometrial carcinoma (EC) is one of the leading causes of female mortality worldwide [1]. Endometrial cancer develops in about 142,000 women worldwide every year, and an estimated 42,000 women die from this cancer. The introduction of ICB (Immune Checkpoint Blockade) has achieved favorable clinical effects in patients with end-stage EC where the chemotherapy regimen has little progression [2, 3]. However, more than 80% of patients are nonresponders, or NDB (no durable clinical benefit), to immunotherapy, and the underlying factors resulting in heterogeneous prognoses are poorly understood. In fact, cancer development and immune response are determined by multiple factors, including genomic mutation [4], DNA methylation [5], and copy number variance [6], et al. Therefore, analysis incorporating multiomics data is urgently needed for EC management.

We utilized meta-dimensional strategies to seek genetically susceptible molecules from gene expression, somatic mutation, copy number alteration, and DNA methylation data of EC, aiming to develop a multiomics signature for prognosis and immunotherapy response of EC. The signature was built by machine learning model, and its efficiency was compared with traditional clinical features. Two computational approaches were also deployed to estimate the signature's performance in predicting immunotherapy response. Further validation focused on the most frequently mutant molecule in the signature: ARID1A. The association of ARID1A with survival, MSI (Microsatellite-instability), immune checkpoints, TIL (tumor-infiltrating lymphocyte), and downstream immune pathways were explored and potential mechanisms was given.

The present study constructed a novel multiomics prognostic signature for prognosis and immunotherapy response of EC, which could guide clinical management of EC and benefit from personalized immunotherapy.

## 2. Methods and Materials

### 2.1. Data Acquisition

Multiomics data of EC (endometrial carcinoma) were acquired from databases, such as the TCGA-UCEC cohort (The Cancer Genome Atlas Endometrial Cancer, 543 tumors, and 35 normal samples) at the UCSC Xena website [7] (https://xenabrowser.net/datapages/).These data included datasets of Copy Number Variation (CNV), DNA methylation (450k), RNA-seq of raw counts, somatic mutation (MuTect2 method), and survival data. In parallel, gene sets of 482 mutated genes with alteration frequency >5% and 380 copy number varied genes with alteration frequency >1% in EC were retrieved from Cbioportal [8] (https://www.cbioportal.org) and OncoKB [9] database (https://oncokb.org).

### 2.2. Differential Expression and Function Enrichment Analysis

To reveal the molecules of real value for EC in these multiomics datasets, a series of *R* packages were used for screening, for example, the limma package [10] to seek out differentially expressed genes between 543 tumor and 35 normal samples with |log2 Fold Change (FC)| > 1.5 and *P* value < 0.05 as the threshold, as well as the ChAMP package [11] to identify differential methylation loci with |log2 Fold Change (FC)| > 0.5 and *P* value < 10^−15^.

A heatmap and volcano plot were used to display the 457 differentially expressed genes (DEG) and 746 CpG sites between tumor and normal samples, with GO (https://wego.genomics.org.cn) and KEGG (https://wego.genomics.org.cn) enrichment analysis to dissect their biological function and related signaling pathways. Meanwhile, oncoprint-plot was employed to present the top 30 mutated and copy number varied genes in EC.

### 2.3. Construction of the Multiomics Prognostic Signature for EC

Subsequent filtration of the 457 significant DEGs, 746 differential methylation loci, 482 mutated, and 380 copy number varied genes was completed by LASSO penalized Cox regression with overall survival as the dependent variable. Finally, 22 molecules were adopted for modeling. Next, Kaplan–Meier curves were depicted to show the prognostic power of the 22-gene-signature where the risk score of each patient was calculated with the following formula: ∑_*i*_^*n*^ Coef_i_^*∗*^*X*_i_ (Coef_i_: cox regression coefficient, *X*_i_: expression value of corresponding molecule, *n* = 22). Following that, patients were stratified into a high- and low-risk group according to the median risk score. A ROC (receiver operator characteristic) curve and multivariate Cox regression were also used to evaluate its prognostic performance and independent prognostic efficiency.

### 2.4. Relationship of the Prognostic Signature with Immunotherapy Response in EC

To assess the relationship of the signature with immunotherapy, algorithms of TIDE [12] (tumor immune dysfunction and exclusion) and Immune Cell AI [13] were applied to predict patients' responses to ICB (immune checkpoint blockade) treatment. A hundred-percent bar-chart and a heatmap were used to display the response difference to ICB between the high and low-risk groups.

### 2.5. Validation on ARID1A for Its Prognostic Ability and Association with Immunotherapy

Further validation focused on the most frequently mutant molecule in the signature: ARID1A. The association of ARID1A mutation with patients' survival, MSI (microsatellite-instability), immune checkpoints or T cell exhaustion markers (LAG3, SIGLEC15, CTLA4, HAVCR2 (TIM3), PDCD1LG2 (PD-L2), CD274 (PD-L1), PDCD1 (PD1), and TIGIT) and downstream immune pathways were explored. In addition, the impact of the ARID1A mutation on the abundances of 22 tumor-infiltrating immune cells was assessed by the CIBERSORT algorithm.

### 2.6. Underlying Mechanism from ARID1A Mutation to Cancer Immune Activation

To identify the underlying mechanism from ARID1A mutation to cancer immune activation, a ternary interaction network was constructed. First, differential expression analysis was carried out between 235 ARID1A-mut samples and 291 ARID1A-wild tumor samples of the UCEC cohort, with 25 upregulated and 46 downregulated DEGs being obtained. By performing correlation analyses between the71 DEGs, abundances of 22 immune cells computed by the CIBERSORT, and enrichment scores of 29 cancer specialized immune pathways [14] quantified by GSVA [15], the interaction pairs of DEG-Immune Cell and DEG-Immune Pathway with a correlation coefficient >0.3 were screened out. A further regulating network of 71 DEG, 22 immune cells, and 29 immune pathways was completed by Cytoscape software (https://cytoscape.org/).

### 2.7. Statistical Analysis

Data processing and all analyses were accomplished by *R* 4.0.4. (Package: limma, ggplot2, survminer, ChAMP, ggcorrplot, GSVA, CIBERSORT, and so on). A chi-square test was used for counting data. Wilcoxon or Kruskal–Wallis tests were applied for comparisons between groups, while the Pearson and Spearman's rank correlation were adopted to estimate the statistical correlation of parametric or nonparametric variables. Two-sided *P* < 0.05 was considered a significant threshold for all statistical tests.

## 3. Results

### 3.1. Differential Expression Analysis between Tumor and Normal Samples

**Figure 1:**
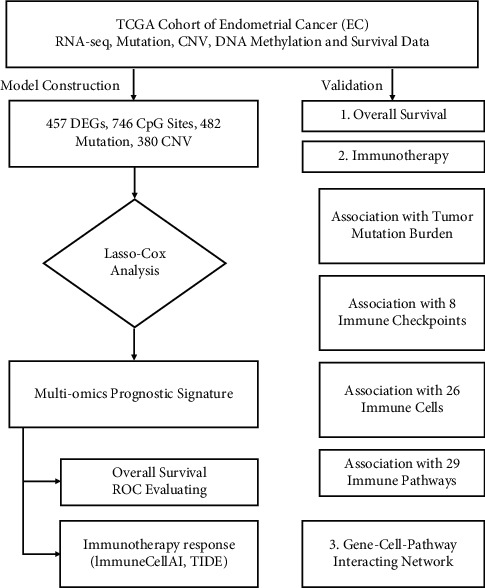
Study protocol.

**Figure 2:**
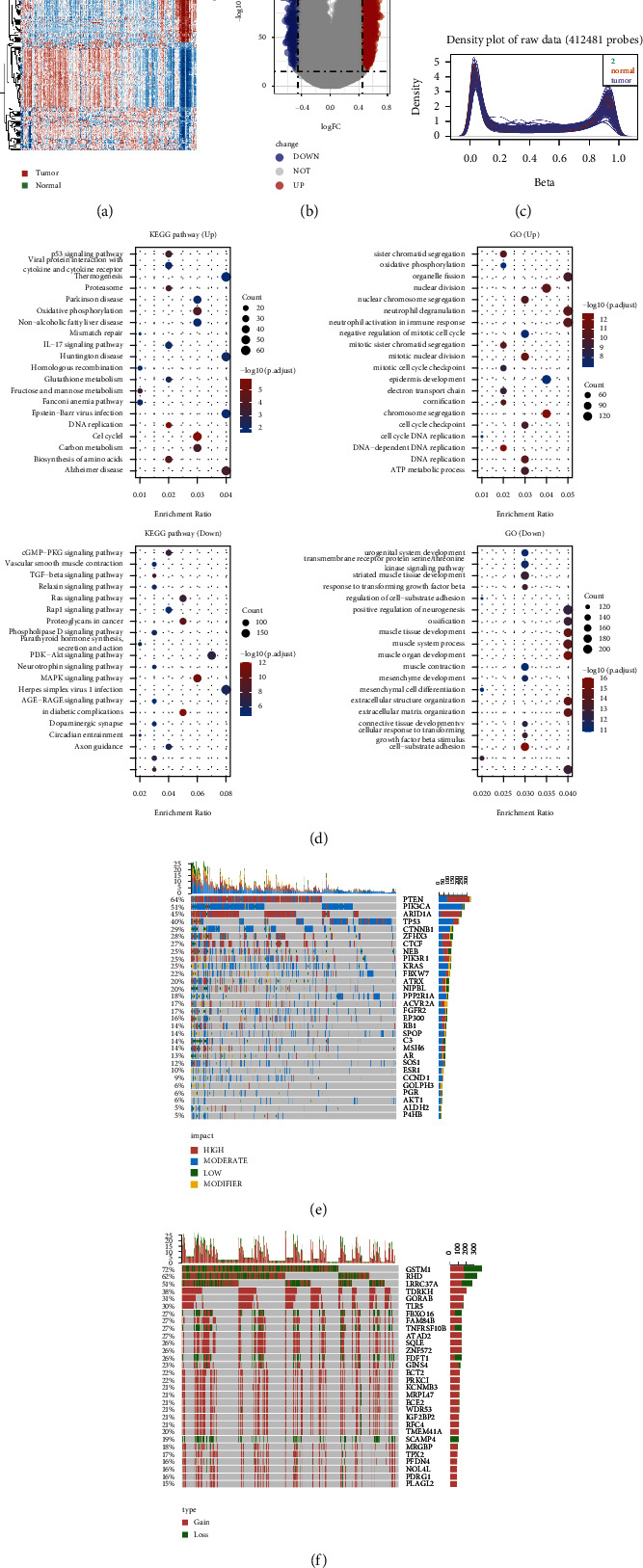
Differential expression and function enrichment analysis. (a) Heatmap of 457 DEGs (differentially expressed genes) between tumor and normal samples. (b) Volcano-plot of 746 differential CpG sites, 3 most upregulated sites marked. (c) Bimodal distribution of Beta value for methylation among tumor and normal samples. (d) KEGG and GO enrichment analysis. (e) Top 30 mutant genes in EC. (f) TOP 30 genes with copy number variance. (UP: upregulated DEGs; DOWN: downregulated DEGs, KEGG : Kyoto Encyclopedia of Genes and Genomes; GO : Gene Ontology).

The study protocol was illustrated in Figure 1 and Table 1 summarized the demographic features of the TCGA-UCEC cohort. 457 differentially expressed genes (DEG) and 746 differential CpG sites are shown in the heatmap and volcano-plot (Figure 2(a)-2(b)). Those DEGs were mainly enriched in thermogenesis and neutrophil activation involved in immune response pathways (Figures 2(c) and 2(d)). The top 30 mutant and copy number varied genes are displayed in the oncoprint-plot (Figures 2(e) and 2(f)).

### 3.2. Construction of the Multiomics Prognostic Signature

**Figure 3:**
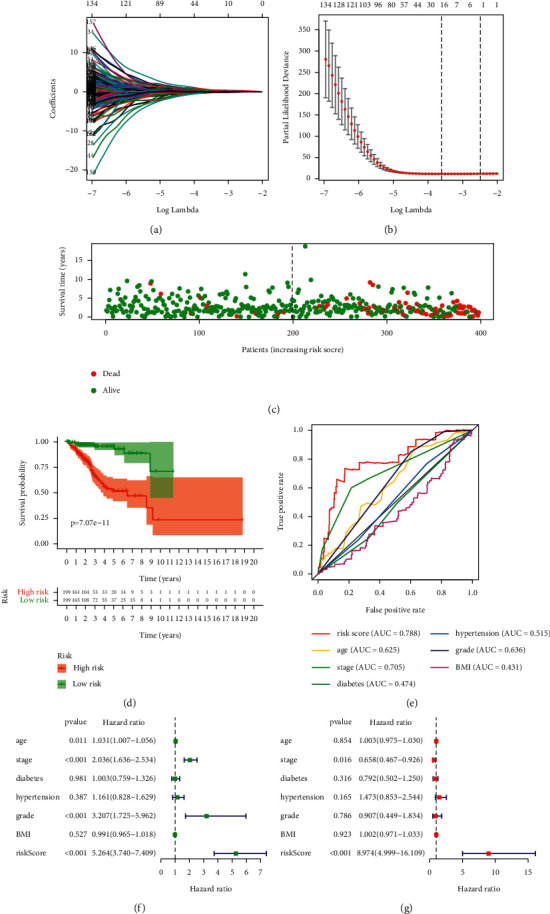
Construction and evaluation of the prognostic signature. (a) Most factors' coefficients were penalized toward zero by LASSO regression. (b) 22 variables were screened out with a minimal partial likelihood deviance. (c) Patients' survival status, ranking by their risk score. (d) Survival analysis between high-risk and low-risk group. (e) Risk score out-weights common clinical features in predicting patients' survival with higher AUC of 0.788. (f) (g) Univariate and multivariate Cox regression demonstrated the prognostic signature can be an independent prognostic factor. (AUC : Area under the curve; BMI : Body mass index).

22 molecules stood out in LASSO-Cox analysis after shrinking most factors' coefficient towards zero (Figure 3(a)-3(b)), including 9 genes with somatic mutation, 4 with copy number variance, 3 with differential CpG sites, and 6 DEGs, their regression coefficients are shown in Table 2. The risk score of each patient was illustrated which well-stratified patients into two groups, according to the median value, with a huge discrepancy in survival probability (Figures 3(c)–3(d)). Patients were illustrated from a database. ROC curve showed a better prognostic performance of the signature than traditional clinical features, such as pathological stage and tumor grade (Figure 3(e)). Subsequent univariate and multivariate Cox analyses proved the signature can be an independent factor for the prognosis of EC (Figures 3(f)–3(g)).

### 3.3. Relationship of the Prognostic Signature with Immunotherapy Response

**Figure 4:**
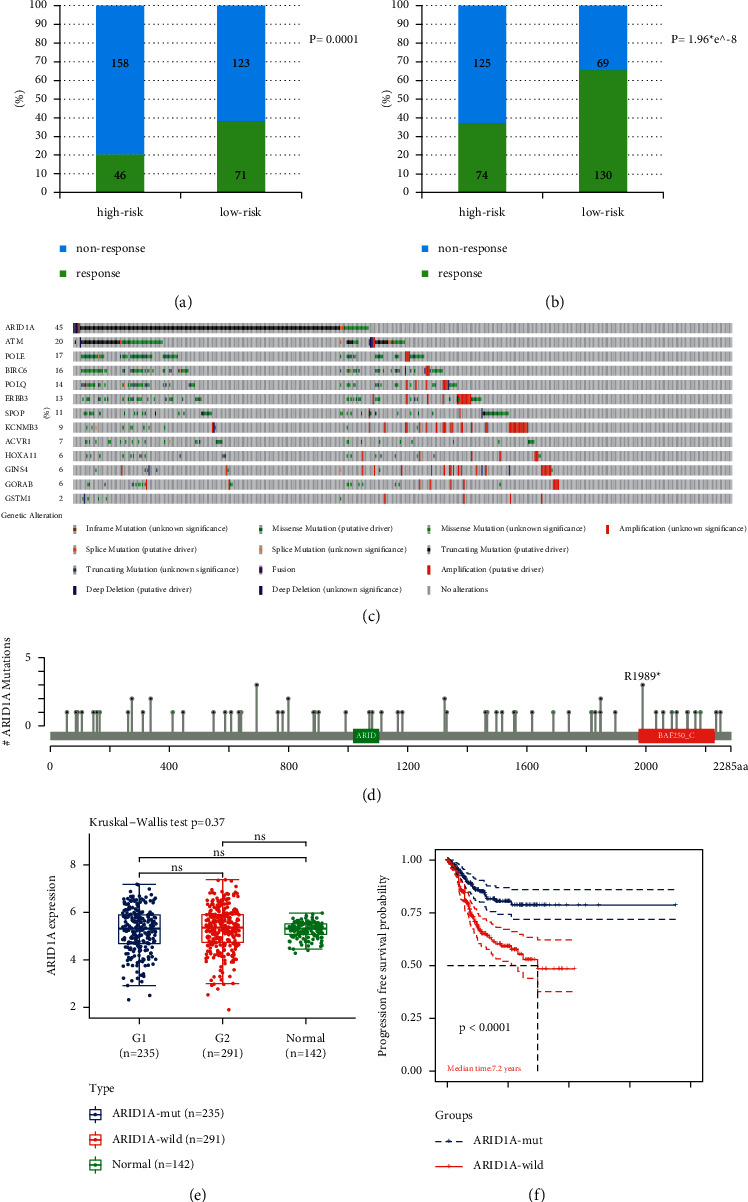
Validation on ARID1A's ability to predict patients' survival outcome. A, b: Difference of immunotherapy response rate between high-risk and low-risk group, predicted by TIDE and Immune cell AI algorithms, respectively. c: Alteration spectrum of 9 mutant and 4 copy number varied genes screened above. d: Mutation sites of ARID1A in EC. e: There were no difference of ARID1A mRNA expression between ARID1A mutant and wild groups. f: ARID1A mutant group showed a better survival outcome in UCEC (Uterine Corpus Endometrial Carcinoma) cohort. (ns: not significant; response and nonresponse: patient response to immunotherapy or vice versa; ARID1A-mut and ARID1A-wild: group with ARID1A mutation or vice versa).

In light of immunotherapy, no matter TIDE or Immune Cell AI algorithm, more patients were seen to be responders to ICB treatment (anti-PD-1 or anti-CTLA4) in the low-risk group than people in the high-risk group (71 vs 46 and 130 vs 74, respectively, *P* < 0.001) with statistically significant difference (Figure 4(a) and 4(b)).

### 3.4. Validation on ARID1A for Its Prognostic Ability and Association with Immunotherapy

**Figure 5:**
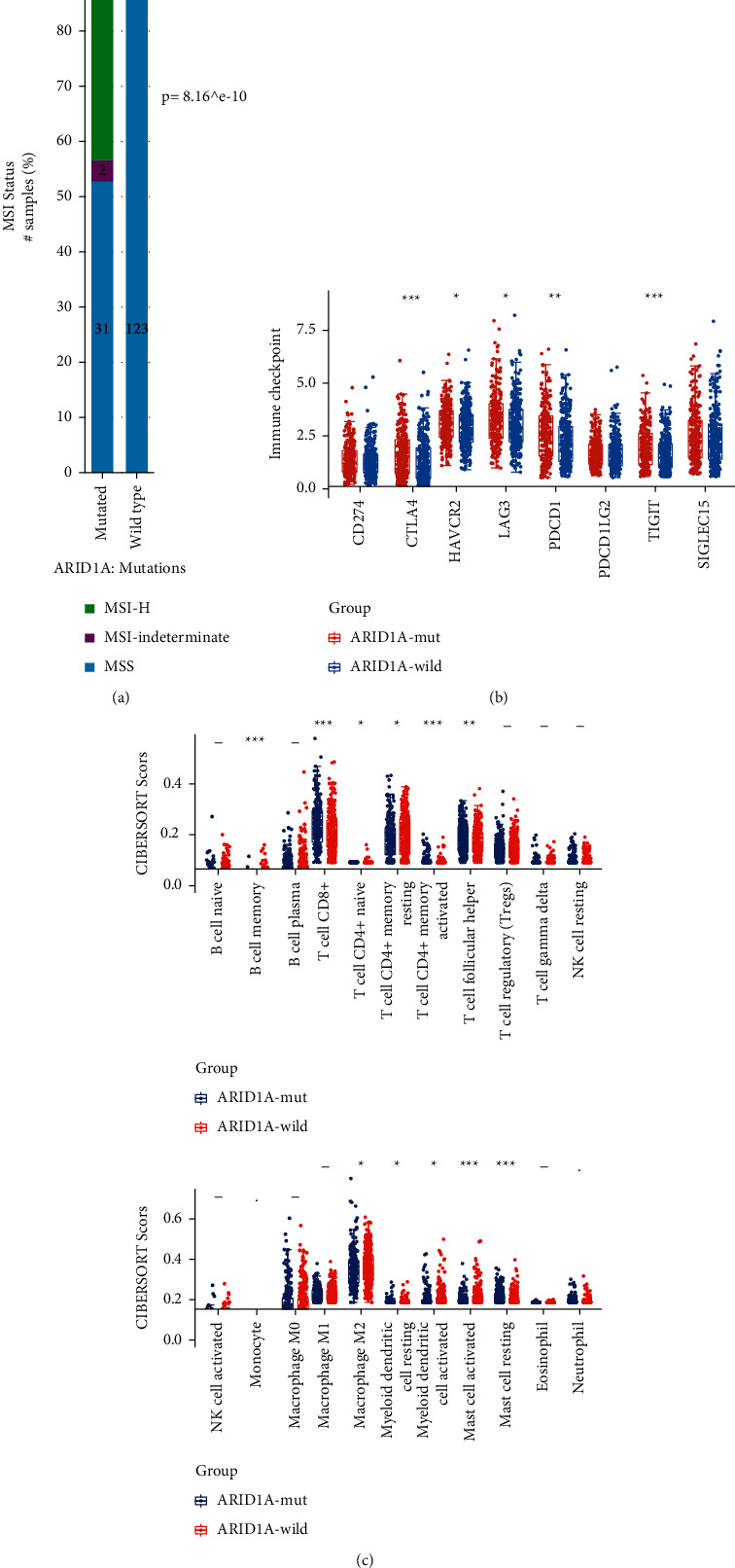
Effect of ARID1A mutation on MSI (microsatellite instability), 8 immune checkpoints and 26 immune cells in EC (endometrial carcinoma). (a) ARID1A mutant group showed higher proportion of MSI-H than wild group in EC. (b) ARID1A mutant group displayed higher level of PDCD1, LAG3, and TIGIT than wild group in EC. (c) ARID1A mutant group exhibited higher infiltration of CD8+ T cell than wild group in EC. (MSI-H : Microsatellite-instability-high; MSS : Microsatellite stability; ^*∗*^: *P* < 0.05; ^*∗∗*^: *P* < 0.01; ^*∗∗∗*^: *P* < 0.001).

As the most frequently mutant gene in EC (Figures 4(c)–4(d), ARID1A can well stratify patients into two groups with noticeable survival differences in the UCEC cohort (4E-4 F), but did not affect their mRNA transcription. ARID1A mutation was also associated with MSI-H status, higher level of immune checkpoints expression, and TIL (tumor-infiltrating lymphocyte) (Figure 5(a)–5(c)).

### 3.5. ARID1A May Interact with Treg and Promote Type-I–IFN–Response Pathway to Facilitate Tumor Immune Activation in EC

**Figure 6:**
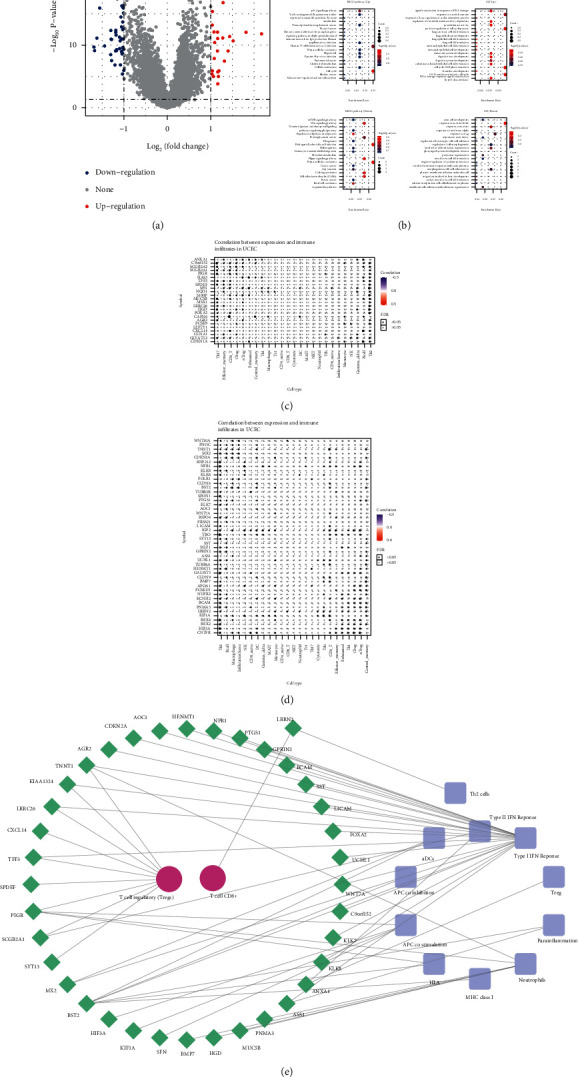
ARID1A mutation may interact with Treg and promote Type-I–IFN–Response pathway to facilitate tumor immune response in EC (endometrial cancer). (a) 71 DEGs (differentially expressed genes), 25 upregulated and 46 downregulated, between ARID1A mutant and wild group in UCEC (uterine corpus endometrial carcinoma) cohort. (b) KEGG and GO enrichment analysis of the 71 DEGs. (c-d) correlation between 25 upregulated genes/46 downregulated genes and abundances of 26 immune cells, respectively. (e) Regulating network between immune pathways (purple), tumor infiltrating cells (red), and DEGs (green). (UCEC : The Cancer Genome Atlas-Uterine Corpus Endometrial Carcinoma cohort; FDR : False Discovery Rate; KEGG : Kyoto Encyclopedia of Genes and Genomes; GO : Gene Ontology).

Of the 71 DEGs, 25 were upregulated and 46 were downregulated between ARID1A mut and ARID1A-wild tumor samples (Figure 6(a)). They were mainly enriched into the p53 signaling, mTOR, DNA damage, and stem cell development signaling pathways (Figure 6(b)). These DEGs also exhibited extensive association with 22 immune cells and 29 immune pathways in the correlation heatmap (Figures 6(c) and 6)(d). Within the final interaction network, the type-I–IFN–Response pathway and T cell regulatory showed a major connection with DEGs, indicating that ARID1A may interact with Treg and promote Type-I–IFN–Response pathway to facilitate tumor immune response in EC (Figure 6)(e).

## 4. Discussion

The present study constructed a novel multiomics prognostic signature for prognosis and immunotherapy response of EC, which could guide clinical management of EC and benefit personalized immunotherapy. Following validation, it indicated the ARID1A mutation may interact with Treg and promote Type-I–IFN–Response pathway to facilitate tumor immune response and better survival outcomes for EC patients.

ARID1A (BAF250a), though connected with a superior outcome of ICB treatment in several cancer types, has rarely been reported for its prognostic and predictive ability in the immunotherapy cohort of EC [16–18]. As a subunit of the SWI/SNF chromatin-remodeling complex, it harbors an N-terminal DNA binding ARID (∼110 residues) and a C-terminal folded region (∼250 residues) [19], which are essential to increasing chromatin accessibility, binding to the promoter regions and facilitating transcription of multiple genes [20]. Inconsistently, the majority of DEGs were found to be downregulated in the ARID1A-mut group in our study (46 vs 25), partly accounting for the tumor suppression effect of ARID1A deficiency in a wide range of cancer types [21–23]. These results were in line with the advantageous role of ARID1A mutation for patients' survival outcomes in the TCGA-UCEC in this study.

In fact, association between ARID1A mutation and favorable ICB treatment outcome in other cancer types is not scarce. Shen *J* et al. have reported a greater proportion of ICB responses in the ARID1A-deficient group than in the ARID1A-wild group in ovarian cancer mouse models [24]. A similar result was also observed in two melanoma cohorts [25–27] (42.86% responders versus 25.81% nonresponders and 100% responders versus 51.43% nonresponders, respectively). In addition, favorable survival outcomes in ARID1A mutant patients when receiving ICB treatment were also revealed in a pan-cancer study [16], but merely 10 EC samples with the ARID1A mutation were included, not sufficient to demonstrate the survival difference.

Elsewhere, ARID1A mutation was seen to be involved in Type-I–IFN–Response pathway and regulatory T cell to interact with EC development, partly accounting for its advantageous role in many kinds of cancer. The previous study has already linked IFN I [28] and IFN II [29] pathway to ICB therapy outcome in multiple cancers and there was data also connecting the ARID1A mutation with IFN I and II Response pathway activity [17]. Apart from IFN pathways, in agreement with our findings, ARID1A mutation could also result in a higher level of PD-1, MSI, and T cell infiltration [30–32] to promote cancer immunity and potentiating favorable ICB treatment response.

Given the inherent fault of bioinformatics analysis-lacking of convincing data from reality. The conclusion of this study may be constrained. Furthermore, multicentric clinical studies and experiments at the cell and animal levels are warranted to validate the results under different circumstances. Following validation, it indicated that ARID1A mutation may interact with Treg and promote Type-I–IFN–Response pathway to facilitate tumor immune response and better survival outcomes for EC patients.

## 5. Conclusion

The present study constructed a novel multiomics prognostic signature for prognosis and immunotherapy response of EC, which could guide clinical management of EC and benefit from personalized immunotherapy.

## Figures and Tables

**Table 1:** Clinical feature of TCGA-UCEC cohort.

	ARID1A-mut	ARID1A-wild	*P*value
SAMPLE	233	288	
AGE	61.49 ± 10.66	65.89 ± 11.02	<0.001
BMI	34.11 ± 15.06	33.54 ± 9.28	0.608
STAGE			0.009
Stage I	164 (70.39%)	159 (55.21%)	
Stage II	21 (9.02%)	29 (10.07%)	
Stage III	44 (18.87%)	77 (26.73%)	
Stage IV	4 (1.72%)	23 (7.99%)	
DIABETES			0.76
NO	120 (74.07%)	138 (72.63%)	
YES	42 (25.93%)	52 (27.37%)	
HYPERTENSION			0.992
NO	75 (42.37%)	84 (42.42%)	
YES	102 (57.63%)	114 (57.58%)	
GRADE			0.025
G1	52 (22.32%)	44 (15.28%)	
G2	58 (24.89%)	57 (19.79%)	
G3	121 (51.93%)	180 (62.50%)	
High grade	2 (0.86%)	7 (2.43%)	
STATUS			<0.001
Alive	213 (91.42%)	223 (77.43%)	
Dead	20 (8.58%)	65 (22.57%)	

**Table 2:** 22 key molecules identified by LASSO-Cox regression.

Molecules	Annotation	Coefficient
ACVR1 (Activin A Receptor Type 1)	Mutation	−0.31351085
ARID1A (AT-Rich Interaction Domain 1A)	Mutation	−0.230538257
ATM (Ataxia Telangiectasia Mutated)	Mutation	−0.095420173
BIRC6 (Baculoviral IAP Repeat Containing 6)	Mutation	−0.13931703
ERBB3 (Erb-B2 Receptor Tyrosine Kinase 3)	Mutation	−0.167684278
HOXA11 (Homeobox A11)	Mutation	0.340669897
POLE (DNA Polymerase Epsilon)	Mutation	−0.18491545
POLQ (DNA Polymerase Theta)	Mutation	−0.035077258
SPOP (Speckle Type BTB/POZ Protein)	Mutation	−0.094758819
GINS4 (SLD5,GINS Complex Subunit 4)	CNV	0.058592508
GORAB (Golgin, RAB6 Interacting)	CNV	0.074299734
GSTM1 (Glutathione S-Transferase Mu 1)	CNV	0.172758754
KCNMB3 (Potassium Calcium-Activated Channel Subfamily M Regulatory Beta Subunit 3)	CNV	−0.111711137
PTPN22 (Protein Tyrosine Phosphatase Non-Receptor Type 22)	DEG	−0.074886487
CDH18 (Cadherin 18)	DEG	0.197447688
KCNK3 (Potassium Two Pore Domain Channel Subfamily K Member 3)	DEG	0.047114247
PCSK1 (Proprotein Convertase Subtilisin/Kexin Type 1)	DEG	0.114882922
KCNJ12 (Potassium Inwardly Rectifying Channel Subfamily J Member 12)	DEG	0.131411471
NCMAP (Non-Compact Myelin Associated Protein)	DEG	−0.024703756
cg07792478	CpG of IR124-2	0.327359364
cg13703871	CpG of NF177	0.583394392
cg14398860	CpG of INPP5A	0.133967149

(CNV, copy number variance; DEG, differentially expressed genes)

## Data Availability

This study was based on secondary databases which are publicly available in the TCGA (https://xenabrowser.net/datapages/)^7^, Cbioportal (https://www.cbioportal.org)^8^, and OncoKB database (https://oncokb.org)^9^, without identification of individual data.

## Abbreviation

EC: Endometrial carcinoma
TCGA-UCEC: The Cancer Genome Atlas-Uterine Corpus Endometrial Carcinoma cohort
ICB: Immune checkpoint blockade
CNV: Copy number variation
MSI: Microsatellite instability
MSI: Microsatellite-instability
TIL: Tumor infiltrating lymphocyte
DEG: Differentially expressed genes
OS: Overall survival
PFS: Progression-Free survival.
